# Analysis of Clinical Characteristics and Virus Strains Variation of Patients Infected With SARS-CoV-2 in Jiangsu Province—A Retrospective Study

**DOI:** 10.3389/fpubh.2021.791600

**Published:** 2021-12-24

**Authors:** Shenjiao Wang, Xin Zou, Zhifeng Li, Jianguang Fu, Huan Fan, Huiyan Yu, Fei Deng, Haodi Huang, Jiefu Peng, Kangcheng Zhao, Lunbiao Cui, LiGuo Zhu, Changjun Bao

**Affiliations:** ^1^Acute Infectious Disease Control and Prevention Institute, Jiangsu Provincial Center for Disease Control and Prevention, Nanjing, China; ^2^School of Public Health, Nanjing Medical University, Nanjing, China; ^3^Institute of Microbiology, Jiangsu Provincial Center for Disease Control and Prevention, Nanjing, China

**Keywords:** SARS-CoV-2, pharyngeal swabs, whole genome sequencing, single nucleotide polymorphisms, spike glycoprotein, clinical characteristics, patients severity SARS-CoV-2, patients severity

## Abstract

**Background:** At present, the global sever acute respiratory syndrome coronavirus 2 (SARS-CoV-2) situation is still grim, and the risk of local outbreaks caused by imported viruses is high. Therefore, it is necessary to monitor the genomic variation and genetic evolution characteristics of SARS-CoV-2. The main purpose of this study was to detect the entry of different SARS-CoV-2 variants into Jiangsu Province, China.

**Methods:** First, oropharyngeal swabs were collected from 165 patients (55 locally confirmed cases and 110 imported cases with confirmed and asymptomatic infection) diagnosed with SARS-CoV-2 infection in Jiangsu Province, China between January 2020 and June 2021. Then, whole genome sequencing was used to explore the phylogeny and find potential mutations in genes of the SARS-CoV-2. Last, association analysis among clinical characteristics and SARS-CoV-2 Variant of Concern, pedigree surveillance analysis of SARS-COV-2, and single nucleotide polymorphisms (SNPs) detection in SARS-COV-2 samples was performed.

**Results:** More men were infected with the SARS-CoV-2 when compared with women. The onset of the SARS-CoV-2 showed a trend of younger age. Moreover, the number of asymptomatic infected patients was large, similar to the number of common patients. Patients infected with Alpha (50%) and Beta (90%) variants were predominantly asymptomatic, while patients infected with Delta (17%) variant presented severe clinical features. A total of 935 SNPs were detected in 165 SARS-COV-2 samples. Among which, missense mutation (58%) was the dominant mutation type. About 56% of SNPs changes occurred in the open reading frame 1ab (ORF1ab) gene. Approximately, 20% of SNP changes occurred in spike glycoprotein (S) gene, such as p.Asp501Tyr, p.Pro681His, and p.Pro681Arg. In total, nine SNPs loci in S gene were significantly correlated with the severity of patients. It is worth mentioning that amino acid substitution of p.Asp614Gly was significantly positively correlated with the clinical severity of patients. The amino acid replacements of p.Ser316Thr and p.Lu484Lys were significantly negatively correlated with the course of disease.

**Conclusion:** Sever acute respiratory syndrome coronavirus 2 (SARS-CoV-2) may further undergo a variety of mutations in different hosts, countries, and weather conditions. Detecting the entry of different virus variants of SARS-CoV-2 into Jiangsu Province, China may help to monitor the spread of infection and the diversity of eventual recombination or genomic mutations.

## Introduction

Severe acute respiratory syndrome coronavirus 2 (SARS-CoV-2), first detected in Wuhan, China, in December of 2019 (1), has spread throughout the world by travel and community-based contacts (2, 3). The SARS-CoV-2 is a pleomorphic, enveloped, positive-sense, and single-stranded RNA virus (4, 5). It has four structural proteins, such as nucleocapsid (N), membrane “matrix” (M), spike (S), and envelope (E). The assembly of these proteins into the infectious virion results in distinct infectivity of these coronaviruses (5–7). Clinically, the majority of SARS-CoV-2 vaccine candidates are based on the S protein (8), which is the major target of neutralizing antibodies (9, 10). Based on the clinical characteristics, patients with SARS-CoV-2 are classified as mild, moderate, severe, and critical (11). Signs and symptoms of SARS-CoV-2 include fatigue, shortness of breath, cough and chest pain, sore throat, respiratory distress, invasive lung lesions, pneumonia with prominent fever, anosmia, and diarrhea (12–18). For severe SARS-CoV-2 disease, major risk factors include age, male sex, smoking, obesity, and comorbid chronic conditions (such as, hypertension, type 2 diabetes mellitus, and others) (19–21). Now, the global SARS-CoV-2 situation is still grim, and the risk of local outbreaks caused by imported viruses is high. Therefore, it is important to monitor the genomic variation and genetic evolution characteristics of SARS-CoV-2. In this study, we collected oropharyngeal swabs from 165 patients (55 locally confirmed cases and 110 imported cases with confirmed and asymptomatic infection) diagnosed with SARS-CoV-2 infection in Jiangsu Province, China between January 2020 and June 2021 to detect the entry of different SARS-CoV-2 variants into Jiangsu Province. Our study could help to monitor the spread of infection and the diversity of genomic mutations in SARS-CoV-2.

## Materials and Methods

### Collection of Samples

Oropharyngeal swabs were collected from 165 patients (locally confirmed cases and imported cases with confirmed and asymptomatic infection) diagnosed with SARS-CoV-2 infection in Jiangsu Province between January 2020 and June 2021. For imported cases, these patients became ill during the 14-day quarantine period, indicating that they were infected abroad. The relevant clinical characteristics of these patients were recorded. According to the SARS-CoV-2 Diagnosis and Treatment Protocol (Trial Version 7) of the National Health Commission, PRC, for course distribution, patients were divided into the following four groups: (1) mild type: the clinical symptoms were mild, and no pneumonia was observed on imaging; (2) common type: patients with fever, respiratory symptoms, and other imaging manifestations of pneumonia, but no dyspnea or other complications; (3) severe type: patients conformed to any of the following: (1) the occurrence of shortness of breath, breathing rate ≥ 30 times/min; (2) at rest, oxygen saturation ≤ 93%; (3) arterial partial pressure of oxygen (PaO_2_)/oxygen concentration (FiO_2_) ≤ 300 mmHg; (4) patients with significant progression > 50% in lung imaging within 24–48 h; (4) asymptomatic type: patients with a positive nucleic acid testing (NAT) result but without any relevant clinical symptoms and radiological changes of the lung during quarantine. All procedures conducted in this study involving human materials were approved by the Ethics Committee of Novel Coronavirus sequence analysis in Jiangsu province (JSJK2021-B016-01). Moreover, all subjects gave written informed consent.

### Whole Genome Sequencing

Total viral RNA was extracted from 200 μl oropharyngeal swabs using the QIAamp Viral RNA Mini Kit (Qiagen, Germany) according to the instructions, and stored at −80°C for further use. Genome-wide specific amplification of total viral RNA was performed using ULSEN® Ultra-sensitive Novel Coronavirus Whole-genome capture Kit (Beijing MicroFuture, V-090418-1, China). PCR products were purified using Agencourt AMPure XP kit (Beckman Coulter, CA, USA). DNA sequencing library was constructed according to the steps of NexteraXT DNA Library Preparation Kit (Illumina, CA, USA). The library was diluted into 100 pmol/μl. The whole genome was deeply sequenced using iseq100 sequencer of Illumina Sequencing platform. The original sequencing data were assembled into a complete sequence using 2019nCOV automatic analysis software of Beijing Micro Future Company. Nucleotide variation sites were compared with reference genome SARS-CoV-2 Wuhan-Hu-1 (GenBank: MN908947.3) using CLC Genomic Workbench 12.0. Mafft software was used for multiple sequence comparison analysis of data from this study (165 cases), public database data on 108 cases (from public SARS-CoV-2 database GISAID, 9 clades and 6 continents), and reference sequences. The comparison results were analyzed by using the MEGA-X software. Neighbor-joining algorithm and Bootstrap were used to construct the phylogenetic tree. Ggtree software was used to mark and beautify the phylogenetic tree. Based on the results of the multi-sequence alignment in the previous step, the assembled virus genome sequence was compared with the reference sequence. The annotation information of loci was based on the general feature format (GFF) annotation information of MN908947.3 and the SNPEFF software. Statistical summaries of variation information and annotation information were performed by using custom Python scripts. For each mutation locus, emphasis was placed on the S gene sequence alignment, single nucleotide polymorphism/insertion/deletion (SNP/INDEL) mutation locus detection, and gene function annotation analysis. Clinical disease progression phenotypes of patients with SARS-CoV-2 were analyzed and summarized and referred to the database of outbreak.info.

## Results

### Basic Clinical Characteristics of the Research Object

Clinical trials of 165 patients with SARS-CoV-2 are shown in Table 1. There were 100 male patients and 65 female patients. Although the number of patients with SARS-CoV-2 decreased in 2021 as compared with that in 2020, there were still more men than women. The onset age of the patients with SARS-CoV-2 in 2021 was 34.55 years, which was younger than that in 2020 (38.55 years). This suggested that the onset of the SARS-CoV-2 showed a trend of younger age. For course distribution, the number of asymptomatic infected patients was large, similar to the number of common patients. The mean infection time of SARS-CoV-2 was 28.22. The infection time in 2021 was 30.2, which was a little longer than that in 2020 (27.5). There were 55 indigenous cases. It is noted that 110 cases came from abroad, such as 49 (49%) cases from Europe and 37 (34%) cases from Asia.

**Table 1:** Clinical features of 165 patients with SARS-COV-2.

	**Totality**	**In 2020**	**In 2021**
**Gender**	165	116	49
Male	100	66	34
Female	65	50	15
**Age (years)**			
Mean value	37.36	38.55	34.55
<20	12	10	2
≤20 <30	51	30	21
≤30 <40	37	25	12
≤40 <50	23	17	6
≤50 <60	23	17	6
≥60	19	17	2
**Course distribution**			
Asymptomatic	65	43	22
Mild	32	24	8
Common	67	49	18
Severe	1	0	1
**Infection time (days)**			
Mean value	28.22	27.5	30.2
**Source region**
Indigenous cases	55	55	0
Imported cases	110	61	49
Asia	37	13	24
Africa	4	0	4
North America	11	6	5
South America	7	2	5
European	49	40	9
Oceania	2	0	2

### Association Between SARS-CoV-2 Variant of Concern and Clinical Characteristics

In addition to GISAID database, there are Pangolin classification A/B main branch, and the descendant branch B.X.X. As shown in Table 2, B.1.1.7 (Alpha), B.1.351 (Beta), and P.1 (Gamma) variants did not appear until March 2021. The B.1.617.2 (Delta) variant did not appear until May 2021. Alpha and Beta variants were mainly from patients in Asia, and Gamma and Delta variants were from patients in South America and Europe. According to the epidemiological characteristics in Table 3, patients infected with Alpha (50%) and Beta (90%) variants were predominantly asymptomatic, while patients infected with Delta (17%) presented severe clinical features.

**Table 2:** Genotype (Pangolin) of SARS-CoV-2 patients at different times and in different regions of origin.

	**Number**	**SARS-CoV-2 variant of concern**			
		**Alpha (B.1.1.7)**	**Beta (B.1.351)**	**Gamma (P.1)**	**Delta (B.1.617.2)**
**Time**		12	10	2	6
From January to June in 2020	0	0	0	0	0
From July to December in 2020	0	0	0	0	0
From January to February in 2021	0	0	0	0	0
From March to April in 2021	13	6	7	1	0
From May to June in 2021	12	6	3	1	6
**Regions of origin**					
Asia	16	6	9	0	1
Africa	1	1	0	0	0
North America	2	1	1	0	0
South America	3	2	0	1	0
Europe	3	2	0	0	1
Oceania	0	0	0	0	0

**Table 3:** Epidemiological characteristics of patients infected with SARS-CoV-2 Variant of Concern.

	**Alpha (B.1.1.7)**	**Beta (B.1.351)**	**Gamma (P.1)**	**Delta (B.1.617.2)**
Gender (Male/Female)	10/2	3/7	1/1	4/2
Age (mean ± standard deviation)	38.0 ± 12.6	34.2 ± 14.2	25	34.5 ± 4.5
**Course distribution**				
Asymptomatic	6	9	1	1
Mild	1	1	0	3
Common	5	0	1	1
Severe	0	0	0	1
Infection time (days)	24-59	10-48	20	24-42

### Pedigree Surveillance of SARS-CoV-2

**Figure 1:**
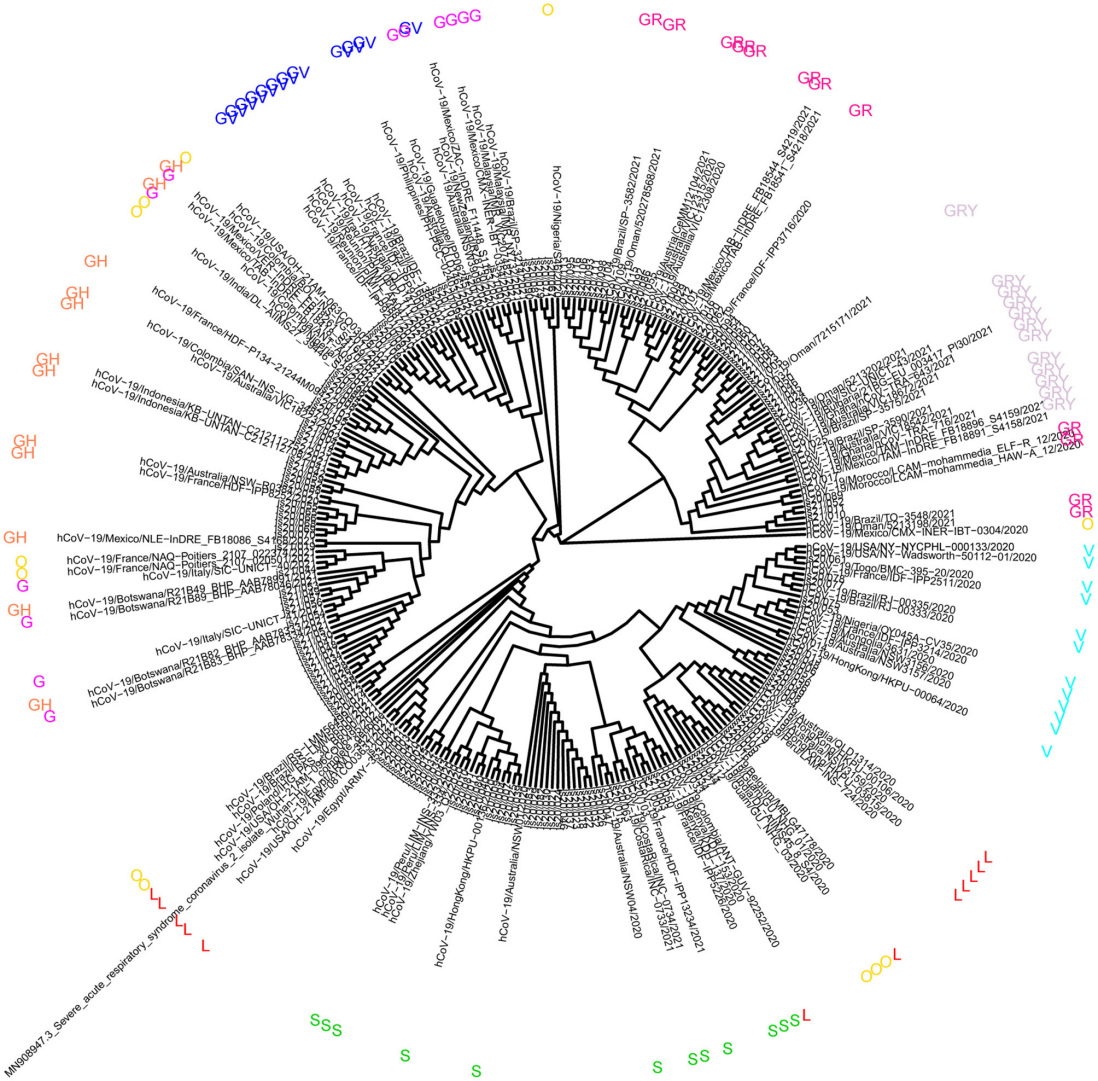
Analysis of phylogeny relationship between the severe acute respiratory symptom of coronavirus 2 (SARSCoV-2) genomes of Coronaviridae of GISAID in Jiangsu Province, China. An interactive view of phylogeny was plotted using Interactive Tree Of Life (iTOL).

The phylogenetic tree was constructed to analyze the mutation regularity and transmission mode of virus evolution to provide the theoretical basis for tracing virus origin and determining virus transmission chain. The 165 sequences described in this study were combined with a phylogenetic tree of 108 genomic datasets (9 clades, 6 continents, and selected 2 cases). According to the GISAID classification, phylogenetic analysis showed that the virus samples from 37 patients were located in clade GR and GRY, 15 patients were located in clade V, 10 patients were from clade S, and 6 patients were located in clade L (Figure 1). Other virus samples were scattered in different clades. It is reported that the high predominance of the clades GR is mainly related to the occurrence of the amino acid substitution p.Asp614Gly that improved viral fitness (22, 23). The global prevalence of variant Alpha has generated clade GRY from clade GR.

### Molecular Analysis of SARS-CoV-2 Samples

**Figure 2:**
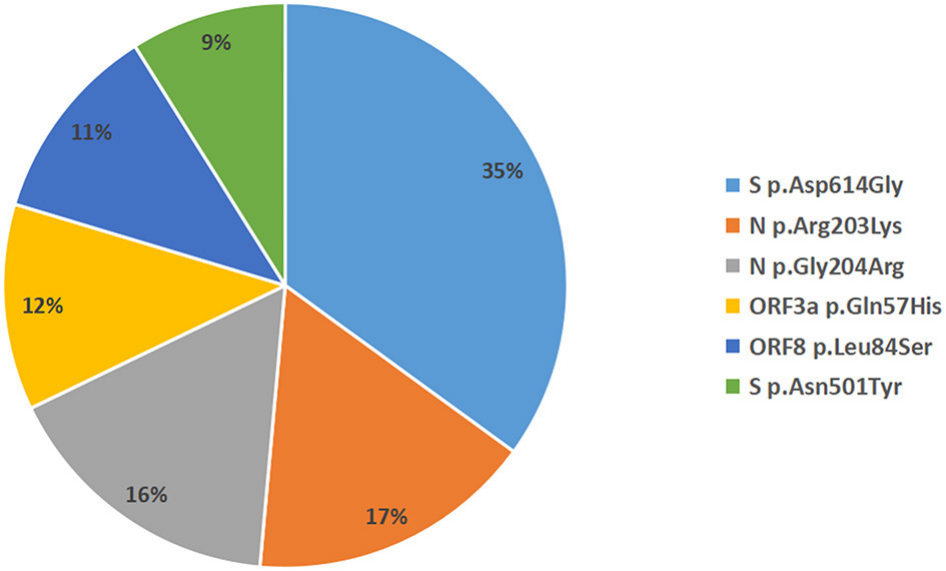
Distribution map of missense mutant genes in 165 SARS-COV-2 samples.

**Figure 3:**
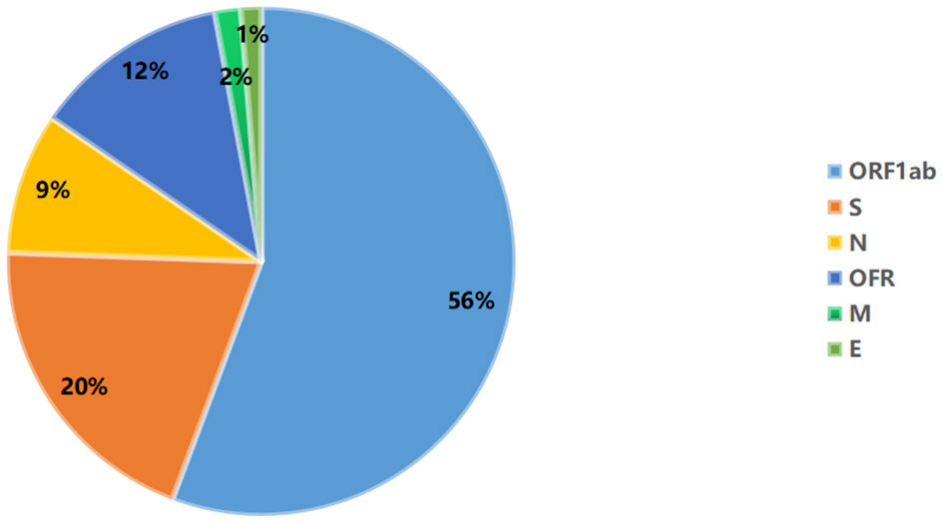
Distribution of amino acid mutation in spike (S), nucleocapsid (N), open reading frame 3a (ORF3a), and open reading frame 3a (ORF8) gene.

A total of 935 single nucleotide polymorphisms (SNPs) were detected in 165 SARS-COV-2 samples, of which 58% of nucleic acid changes resulted in amino acid substitution (missense mutations) (Supplementary Table 1). In the missense mutation, 303 (56%) SNP changes occurred in the open reading frame 1ab (ORF1ab) gene and 108 (20%) SNP changes occurred in the S gene (Figure 2). As showed in Figure 3, two amino acid substitutions occurred in N gene, such as p.Gli204Arg substitution in 46 SARS-COV-2 samples (16%) and p.Arg203Lys in 46 SARS-COV-2 samples (17%). The p.Glin57His substitution in open reading frame 3a (ORF3a) and the p.Leu84Ser substitution in open reading frame 8 (ORF8) was found in 33 (12%) and 32 (11%) SARS-COV-2 samples, respectively. In S gene, 98 SARS-COV-2 samples (35%) had p.Asp614Gly substitution and 25 SARS-COV-2 samples (9%) had p.Asp501Tyr substitution. In addition to the above mutations, amino acid substitutions of p.Pro681His and p.Pro681Arg were found in the S gene in 27 and 27 SARS-COV-2 samples, respectively (Table 4). In addition, Pearson's correlation coefficient was used to analyze the correlation between SNP changes in the S gene and the severity of patients. Interestingly, 9 SNPs loci in S gene were found to be significantly correlated with severity of patients (Supplementary Table 2). Among which, the amino acid substitution of p.Asp614Gly (cor = 0.29, *p* < 0.001) was significantly positively correlated with the clinical severity of patients. The amino acid replacements of p.Ser316Thr (cor = −0.16, *p* = 0.04) and p.Lu484Lys (cor = −0.28, *p* < 0.001) were significantly negatively correlated with the course of disease.

**Table 4:** Main amino acid mutations in S gene in patients infected with SARS-COV-2.

**Amino acid mutations**	**Number**	**Common**	**Mild**	**Asymptomatic**
V70F	0	0	0	0
A222V	9	1	0	8
W258L				
K417N	10	0	1	9
E484K	14	1	1	12
E484Q				
L452R	9	3	3	1
L452Q	9	3	3	1
N501Y	25	7	2	16
D614G	98	32	12	53
P681H	27	10	6	10
P681R	27	10	6	10

## Discussion

According to the basic clinical characteristics of infected patients in Jiangsu Province, China, we found that the onset age of the patients with SARS-CoV-2 in 2021 was 34.55 years, which was younger than that in 2020 (38.55). This suggested that the onset of the SARS-CoV-2 showed a trend of younger age. Moreover, we found that there were still more male patients than female patients, even though the number of SARS-CoV-2 patients decreased in 2021 compared with that in 2020. A study has reported the association between SARS-COV-2 infection and transmission and gender (24). In addition, Jin et al. found that gender could be an important factor affecting the SARS-COV-2 mortality (25). It is assumed that gender differences in susceptibility toward SARS-CoV-2 may be associated with the immune response (26). The data of 10 European countries on the distribution of SARS-COV-2 cases by gender showed that women of working age outnumbered infected men (27). Interestingly, it is reported that male sex is one of the major risk factors for severe SARS-CoV-2 disease (19–21). This is indicated that men could be more likely to develop SARS-CoV-2 than women. In addition, mean infection time of SARS-CoV-2 was 28.22. The infection time in 2021 was 30.2, which was a little longer than that in 2020 (27.5). Furthermore, the number of asymptomatic infected patients was large, similar to common patients. SARS-CoV-2 causes asymptomatic infections. Asymptomatic infection is defined as an individual with a positive NAT result but without any relevant clinical symptoms and radiological changes of the lung during quarantine. It is found that the number of SARS-CoV-2 infections is still rising rapidly, and asymptomatic infections could play roles in transmission (28). The higher asymptomatic infection of SARS-CoV-2 could be associated with the high replication efficiency of the virus (29, 30). Therefore, it is urgent to timely detect the source of asymptomatic infections to provide scientific data as a reference to fight against SARS-CoV-2. Among all patients, we found that 110 cases came from abroad, such as 49 (49%) cases from Europe and 37 (34%) cases from Asia. Thus, it is also important to strictly control the importation of the virus abroad.

Based on the novel coronavirus genome information currently published worldwide, mutation rate of SARS-CoV-2 is about 1~2 nucleotides/month. On February 25, 2021, the WHO first proposed the definition of SARS-COV-2 Variant of Concern. As of 24 June 2021, 4 SARS-COV-2 Variant of Concern have been defined, such as B.1.1.7 (Alpha), B.1.351 (Beta), P.1 (Gamma), and B.1.617.2 (Delta). Variant of Concern, Alpha, is first detected in southeast England in September 2020 and spread to become the dominant lineage in the United Kingdom. Alpha variant has been spreading to at least 114 countries worldwide. The Alpha variant is characterized by 17 mutations. The Beta variant was isolated from an oropharyngeal swab from a patient in the Ugu district, South Africa in November 2020. The emergence of the Beta variant includes several mutations within the structural and nonstructural proteins (31). It is found that variant Beta is purported to be more transmissible (32). The Gamma variant (acquired 17 mutations) emergence occurred around mid-November 2020 in Brazil and was preceded by a period of faster molecular evolution. Gamma variant is purported to be more transmissible (33). In April 2021, the Delta variant was identified in India and classified on May 11, 2021 as a Variant of Concern due to its fast spreading and potential immune escape (34). The Delta variant has been suggested to be more transmissible than the Alpha variant, which is more transmissible than the wild-type SARS-CoV-2 virus (35, 36). Delta variant has now been detected across the Europe, such as the United Kingdom. In our study, we found that Alpha, Beta, and Gamma variants did not appear until March 2021. Delta variant did not appear until May 2021. Tracing its origin, we found that Alpha and Beta variants were mainly from patients in Asia, and Gamma and Delta variants were from patients in South America and Europe. It is worth mentioning that patients infected with Alpha and Beta variants were predominantly asymptomatic, while patients infected with Delta variant presented severe clinical features. The abovementioned variants have increased transmissibility or harmful changes in epidemic characteristics, increased virulence, or clinical manifestations. Therefore, increased surveillance and strict control of the importation of the virus remain urgent.

The SARS-CoV-2 genome shows the typical structure of coronavirus, 5' Untranslated regions (UTR), genes of ORF1ab, ORF3a, ORF8, N, S, E, Membrane protein (M) and 3' UTR containing poly (A) tail, and several unidentified unstructured ORFs (37). It is noted that the mutation rate is fastest at the ORF1ab, ORF3a, and ORF8, N and S genes (38, 39). In the present study, a total of 935 SNPs were detected in 165 SARS-COV-2 samples in Jiangsu Province, China. Missense mutation (58%) was the dominant mutation type. Among which, 56% of SNP changes occurred in ORF1ab gene. ORF1ab, encodes a large polyprotein pp1ab, is proteolytically cleaved into 16 non-structural proteins critical for viral replication (40). In addition, 20% of SNP changes occurred in the S gene, such as p.Asp501Tyr, p.Pro681His, and p.Pro681Arg. It is worth mentioning that p.Asp614Gly variant was significantly positively correlated with the clinical severity of patients. The p.Ser316Thr and p.Lu484Lys variants were significantly negatively correlated with the course of disease. Around September 2020, genetic variants carrying the p.Asp501Tyr substitution on the S protein of SARS-CoV-2 were first detected in the United Kingdom, and spread to elsewhere globally, such as Brazil, South Africa, and the United States (41–44). The p.Asp501Tyr substitution reduces the neutralization capacity of antibodies generated against wild type virus (45–47). Detection of p.Asp501Tyr mutation flags samples that may harbor mutations associated with immune evasion and increased transmissibility. The p.Pro681His mutation is the characteristic of the new SARS-CoV-2 variants from the United Kingdom and Nigeria. The p.Pro681His mutation has unique and emerging characteristics with a significant exponential increase in worldwide frequency. Previous SARS-CoV-2 studies suggest that the p.Pro681His mutation is associated with enhanced systemic infection and increased membrane fusion (48–50). The p.Pro681Arg mutation, was first identified in Uganda in September 2020, is designated a variant of interest in Africa and with subsequent spread to other countries. The p.Pro681Arg mutation causes a small increase in proteolytic processing that might have an effect on viral replication, transmissibility, or pathogenic properties (51). The variant p.Asp614Gly is associated with increased viral load in patients with SARS-CoV-2 (52–54), which may significantly enhance the infectivity and transmissibility of the virus. The mutation p.Lu484Lys, first identified in March 2020, has now been identified in the United Kingdom fast-spreading variant, prompting fears that the virus is evolving further and could become resistant to vaccines (55). The p.Lu484Lys mutation is associated with the humoral immune response evasion (56), which may affect antibody neutralization. This indicated that abovementioned variants in S gene are associated with poor clinical outcome of patients with SARS-CoV-2.

## Conclusion

Our study provided crucial epidemiological data on the clinical features, variation characteristics of SARS-CoV-2 in Jiangsu Province, China. These findings could help to assess the burden of viral infection in patients. Timely and accurate monitoring of the spreading of infection and the diversity of genomic mutations in SARS-CoV-2 are needed to alleviate the burden of these diseases. There are limitations to our study. First, the small number of samples may bring some errors, therefore, larger numbers of samples are further needed to minimize the errors. Second, potential biological function of variants in genes is further needed to investigate. Anyway, our findings could have certain reference value for the evaluation of patients with SARS-CoV-2 and clinical treatment.

## Data Availability Statement

The datasets presented in this study can be found in online repositories. The names of the repository/repositories and accession number(s) can be found in the article/Supplementary Material.

## Ethics Statement

All procedures conducted in this study involving human materials were approved by the Ethics Committee of Novel Coronavirus sequence analysis in Jiangsu province (JSJK2021-B016-01). Moreover, all subjects gave written informed consent. The patients/participants provided their written informed consent to participate in this study.

## Author Contributions

SW, LZ, and CB: conception and design. LZ, CB, and LC: administrative support. SW, ZL, JF, HF, HY, FD, KZ, and LC: provision of materials and samples. SW, XZ, ZL, JF, HF, HY, FD, HH, and JP: data collection and collation. SW and XZ: data analysis and interpretation. All author read and approved the final manuscript.

## Funding

This study was funded by the Social Development Project of Jiangsu Province (BE2021739, provided by LZ).

## Conflict of Interest

The authors declare that the research was conducted in the absence of any commercial or financial relationships that could be construed as a potential conflict of interest.

## Publisher's Note

All claims expressed in this article are solely those of the authors and do not necessarily represent those of their affiliated organizations, or those of the publisher, the editors and the reviewers. Any product that may be evaluated in this article, or claim that may be made by its manufacturer, is not guaranteed or endorsed by the publisher.
